# Characterization of the complete mitochondrial genome of *Blaps rynchopetera* Fairmaire (Insecta: Coleoptera: Tenebrionidae) from Dali

**DOI:** 10.1080/23802359.2019.1667905

**Published:** 2019-09-23

**Authors:** Yang Yang, Yu Bai, Jun Zheng, Jun Chen, Bocheng Ouyang, Sheng Liang

**Affiliations:** a College of Mathematics & Information Science, Guiyang University, Guiyang, China;; b Guizhou Provincial Key Laboratory for Rare Animal & Economic Insects of the Mountainous Region, Guiyang University, Guiyang, China;; c School of Electronic & Communication engineering, Guiyang University, Guiyang, China

**Keywords:** *Blaps rynchopetera*, Tenebrionidae, medicinal insect, mitochondrial genome, resource insect

## Abstract

The complete mitochondrial genome of *Blaps rynchopetera* Fairmaire (GenBank accession number MN267802) from Dali city consists of a circular DNA molecule of 16,766 bp (with 72.01% A + T content), which is longer than that of the mitogenome of *B. rynchopetera* from Kunming. The mitogenome comprises 13 protein-coding, 22 tRNA, and 2 rDNA genes. The protein-coding genes have typical ATN (Met) initiation codons, and are terminated by typical TAN stop codons. A phylogenetic tree generated by the neighbour-joining method showed that *B. rynchopetera* is closely related to *Platydema* sp. PLA01, which has the potential for medicinal development.

*Blaps rynchopetera* Fairmaire is an important medicinal insect used in traditional Chinese medicine. There is only one other mitochondrial sequence of *B. rynchopetera*, which was obtained from Kunming (Zhao et al. 2019). Here, we report the characterization of the complete mitogenome of *B. rynchopetera* from Dali city to obtain more comprehensive biological insight into the population genetics of *B. rynchopetera*.

Samples of adult *B. rynchopetera* (GYU-20180723-001) were obtained from Dali city (E100°23′, W25°60′), Yunnan Province, China on July 23, 2018. Genomic DNA was isolated and fragmented to build a genomic library that was sequenced (paired end 2 × 150 bp) using an Illumina HiSeq 4000. We obtained approximately 11,494 Mbp of raw data and 10,751 Mbp of high-quality, clean data (93.54%). The genome was assembled *de novo* with NOVOPlasty software, version 3.2 (https://github.com/ndierckx/NOVOPlasty) (Dierckxsens et al. 2016).

The mitogenome of *B. rynchopetera* consists of a 16,766 bp circular DNA molecule, with 41.64% A, 31.52% T, 16.53% C, and 10.31% G, which has an A/T bias (72.01% A + T content) and is longer than the *B. rynchopetera* mitogenome of the Kunming population. The AT- and GC-skews of the major strands of the mitogenome were calculated to be approximately 0.1383 and 0.2316, respectively. The length of the A/T-rich region in the mitogenome is 1873 bp, with 75.23% A + T content, and is located between the srRNA and tRNA Ile.

The mitogenome of *B. rynchopetera* contains 13 protein-coding genes (PCGs) and 22 tRNA and 2 rRNA genes. All 13 PCGs have typical ATN (Met) start codons and TAN stop codons; three genes (*cox1*, *cox2*, and *atp8*) have ATA as a start codon, four genes (*nad3*, *nad6*, *nad5*, and *nad1*) have ATT as a start codon, and six genes (*nad2*, *atp6*, *cox3*, *nad4*, *nad4l*, and *cob*) have ATG as a start codon. Seven genes (*nad2*, *cox1*, *cox2*, *atp6*, *nad4l*, *nad6*, and *cob*) have a TAA stop codon; three genes (*atp8*, *nad3*, and *nad1*) had a TAG stop codon, and three genes (*nad4*, *nad5* and *cox3*) have an incomplete stop codon consisting of a T, which is completed by the addition of 3′A nucleotides to the resultant mRNA. The 22 tRNA genes are interspersed throughout the coding region and range from 60 (trnS1) to 70 bp (trnK) in length. The A + T content of tRNA genes ranges from 64.06% (trnY) to 89.39% (trnE). The size of the lrRNA and srRNA genes in the *B. rynchopetera* mitogenome were 1225 and 761 bp long, with 76.65% and 75.82% A + T, respectively.

To validate the phylogenetic position of *B. rynchopetera*, the mitogenome DNA sequences from 16 species of Tenebrionidae were used to construct a phylogenetic tree by the neighbour-joining method using the MEGA X version 10.0.0 software (Kumar et al. 2018) (Figure 1). *B. rynchopetera* was closely clustered with *Platydema* sp. PLA01, which has the potential for medicinal development. In conclusion, our study provides biological insight into the mitogenome of *B. rynchopetera* and its population structure, which will be useful for the utilization and development of this important resource insect.

**Figure 1.**
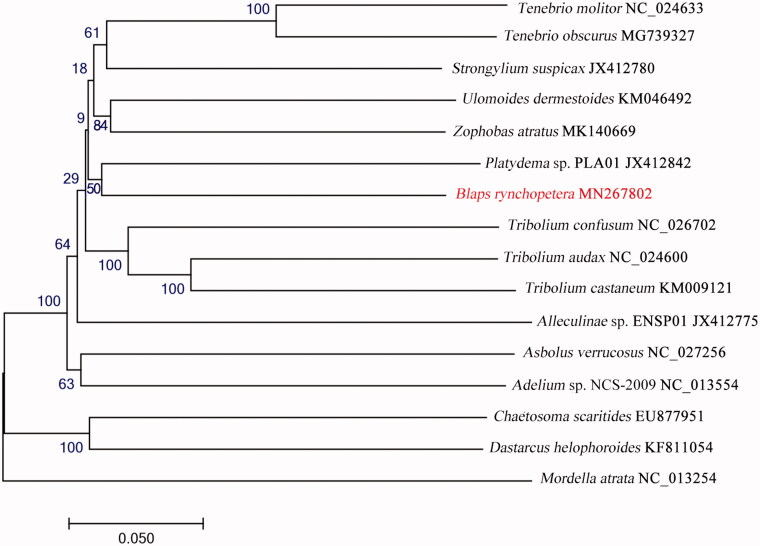
The neighbour-joining phylogenetic tree of *B. rynchopetera* and other 15 beetles of Tenebrionidae based on the DNA sequences of mitogenome.
